# Supplementary material for: A Critical View of the Application of the APEX Software (Aqueous Photochemistry of Environmentally-Occurring Xenobiotics) to Predict Photoreaction Kinetics in Surface Freshwaters
Source: Molecules. 2019 Dec 18;25(1):9. doi: 10.3390/molecules25010009 (PMC7017383; doi:10.3390/molecules25010009)
Supplement: Supplementary file 1 [file molecules-25-00009-s001.zip › 3.2.4_gcc-4.4.0.zip!/3.2.4_gcc-4.4.0/3.2.4_gcc-4.4.0/doc/octave/html/interpreter/Getting-Started-with-Mex_002dFiles.html]

Getting Started with Mex-Files - Untitled


Next: Working with Matrices and Arrays in Mex-Files,
Up: Mex-Files


---

#### A.2.1 Getting Started with Mex-Files

The basic command to build a mex-file is either `mkoctfile --mex` or
`mex`. The first can either be used from within Octave or from the
command line. However, to avoid issues with the installation of other
products, the use of the command `mex` is limited to within Octave.

— Function File: **mex** [options] file ...  
> Compile source code written in C, C++, or Fortran, to a MEX file.
> This is equivalent to `mkoctfile --mex [options] file`.
>
>
>
> **See also:** mkoctfile.

— Function File: **mexext** ()  
> Return the filename extension used for MEX files.

One important difference between the use of mex with other products and
with Octave is that the header file "matrix.h" is implicitly included
through the inclusion of "mex.h". This is to avoid a conflict with the
Octave file "Matrix.h" with operating systems and compilers that don't
distinguish between filenames in upper and lower case

Consider the short example

```
```
     #include "mex.h"
     
     void
     mexFunction (int nlhs, mxArray *plhs[], int nrhs, 
     	     const mxArray *prhs[])
     {
       mxArray *v = mxCreateDoubleMatrix (1, 1, mxREAL);
       double *data = mxGetPr (v);
       *data = 1.23456789;
       plhs[0] = v;
     }
```
```

This simple example demonstrates the basics of writing a mex-file. The
entry point into the mex-file is defined by `mexFunction`. Note
that the function name is not explicitly included in the
`mexFunction` and so there can only be a single `mexFunction`
entry point per-file. Also the name of the function is determined by the
name of the mex-file itself. Therefore if the above function is in the
file firstmexdemo.c, it can be compiled with

```
     mkoctfile --mex firstmexdemo.c
```

which creates a file firstmexdemo.mex. The function can then be run
from Octave as

```
     firstmexdemo()
     => 1.2346
```

It should be noted that the mex-file contains no help string for the
functions it contains. To document mex-files, there should exist an
m-file in the same directory as the mex-file itself. Taking the above as
an example, we would therefore have a file firstmexdemo.m that might
contain the text

```
     %FIRSTMEXDEMO Simple test of the functionality of a mex-file.
```

In this case, the function that will be executed within Octave will be
given by the mex-file, while the help string will come from the
m-file. This can also be useful to allow a sample implementation of the
mex-file within the Octave language itself for testing purposes.

Although we cannot have multiple entry points into a single mex-file,
we can use the `mexFunctionName` function to determine what name
the mex-file was called with. This can be used to alter the behavior of
the mex-file based on the function name. For example if

```
```
     #include "mex.h"
     
     void
     mexFunction (int nlhs, mxArray *plhs[], int nrhs, 
     	     const mxArray *prhs[])
     {
       const char *nm;
       nm = mexFunctionName ();
       mexPrintf ("You called function: %s\n", nm);
       if (strcmp (nm, "myfunc") == 0)
         mexPrintf ("This is the principal function\n", nm);
       return; 
     }
```
```

is in file myfunc.c, and it is compiled with

```
     mkoctfile --mex myfunc.c
     ln -s myfunc.mex myfunc2.mex
```

Then as can be seen by

```
     myfunc()
     => You called function: myfunc
         This is the principal function
     myfunc2()
     => You called function: myfunc2
```

the behavior of the mex-file can be altered depending on the functions
name.

Allow the user should only include `mex.h` in their code, Octave
declares additional functions, typedefs, etc., available to the user to
write mex-files in the headers `mexproto.h` and `mxarray.h`.
